# Genetics of Endometrial Cancers

**DOI:** 10.1155/2010/984013

**Published:** 2010-04-08

**Authors:** Tsuyoshi Okuda, Akihiko Sekizawa, Yuditiya Purwosunu, Masaaki Nagatsuka, Miki Morioka, Masaki Hayashi, Takashi Okai

**Affiliations:** ^1^Department of Obstetrics and Gynecology, Showa University School of Medicine, 1-5-8 Hatanodai, Shinagawa-ku, Tokyo 142-8666, Japan; ^2^Department of Obstetrics and Gynecology, University of Indonesia, Cipto Mangunkusumo National Hospital, Jakarta, Indonesia

## Abstract

Endometrial cancers exhibit a different mechanism of tumorigenesis and progression depending on histopathological and clinical types. The most frequently altered gene in estrogen-dependent endometrioid endometrial carcinoma tumors is *PTEN*. Microsatellite instability is another important genetic event in this type of tumor. In contrast, *p53* mutations or Her2/neu overexpression are more frequent in non-endometrioid tumors. On the other hand, it is possible that the clear cell type may arise from a unique pathway which appears similar to the ovarian clear cell carcinoma. *K-ras* mutations are detected in approximately 15%–30% of endometrioid carcinomas, are unrelated to the existence of endometrial hyperplasia. A *β*-*catenin* mutation was detected in about 20% of endometrioid carcinomas, but is rare in serous carcinoma. Telomere shortening is another important type of genomic instability observed in endometrial cancer. Only non-endometrioid endometrial carcinoma tumors were significantly associated with critical telomere shortening in the adjacent morphologically normal epithelium. Lynch syndrome, which is an autosomal dominantly inherited disorder of cancer susceptibility and is characterized by a MSH2/MSH6 protein complex deficiency, is associated with the development of non-endometrioid carcinomas.

## 1. Introduction

Endometrial cancer is the most common cancer of the female reproductive tract with 150,000 new cases diagnosed annually worldwide. Approximately 90% of endometrial cancers are sporadic, and the remaining 10% are hereditary. Bokhman have generally categorized endometrial cancer into two broad groups of tumors using both clinical and histopathological variables: estrogen-dependent endometrioid endometrial carcinomas (EECs), or type I, and non-endometrioid endometrial carcinomas (NEECs), or type II tumors (Table 1) [1]. It should be noted that this model is not strict, and only a minority of endometrial cancer may exhibit shared characteristics. For example, mixed serous and endometrioid tumors are being increasingly recognized. Approximately 70% to 80% of new cases are classified as EECs, and other 10% to 20% are designated as NEEC tumors [1]. EECs are strongly associated with the estrogen-related pathway and arise in association with unopposed estrogen stimulation [2]. In contrast, NEECs are unrelated to the estrogen pathways and arise in the background of atrophic endometrium [3]. EECs typically occur in premenopausal and younger postmenopausal women and are usually low-grade and have a favorable outcome, whereas NEECs occur in older postmenopausal women. In addition, NEECs tend to predict a high tumor grade and poor patient prognosis [4, 5]. The first pathway is associated with endometrioid histopathology, and the second is linked to the serous and clear cell subtypes. The precursors of these subtypes are known as atypical hyperplasia and endometrial intraepithelial carcinoma (EIC), respectively. Clear cell cancer, classified as an NEEC, is associated with atypical hyperplasia as well as EIC. 

Recent reports suggest that histological differences may be associated with distinct molecular genetic alterations. Molecular genetic evidence indicates that endometrial carcinomas are likely to develop as the result of a multistep process of oncogenic activation and tumor suppressor inactivation (Table 2) [6].

## 2. Gain-of-Function Genetic Events

The genes implicated in the gain-of-function events are oncogenes. The important genes related to endometrial oncogenesis or progressions are the *K-ras*,*B-raf, Her2/neu*, *β* *-catenin, AKT*, and *FGFR2* oncogenes.

### 2.1. *K-ras* and *B-raf*

*K-ras* proto-oncogene mutations are detected in approximately 10%–30% of endometrioid carcinomas [7]. *K-ras* mutations have been identified in endometrial hyperplasias, although at a lower frequency than in carcinomas [8–10]. According to these studies, the gain of the *K-ras* function may represent an early event in endometrioid-type tumorigenesis. During tumorigenesis, activated RAS is usually associated with enhanced proliferation, transformation, and cell survival. Conversely, *K-ras* mutations occur with equal frequency in tumors with and without hyperplasia, and the epidemiologic results seem to suggest that *K-ras* activation is associated with malignant progression of endometrial tumors without the need for transition via hyperplasia [11]. In contrast to endometrioid carcinomas,* K-ras* mutations are extremely rare among serous and clear cell carcinomas [12, 13]. 

A correlation between colon cancer development and Ras/Raf point mutations in the MAP kinase pathway drives the malignant transformation of colon cancer. In contrast, only a few reports have shown *B-raf* mutations in patients with endometrial cancer. Feng et al. identified a *B-raf* mutation in 21% of patients with endometrial cancers and suggest that the mutation correlated with decreased hMLH1 expression [14]. In contrast, Salevesen et al. described a *B-raf *mutation in only 2% of endometrial cancers; and Kawaguchi et al. and Mizumoto et al. reported no mutation in the patients with endometrial cancer [15–17]. Therefore, a consensus about the role of *B-raf* mutation in the development of endometrial cancer has not yet been developed.

### 2.2. Her2/neu

*Her2/neu (erbB2)* is an oncogene that encodes a transmembrane receptor tyrosine kinase involved in cell signaling. Either the overexpression or gene amplification of *Her2/neu* proto-oncogene activates receptor and soluble tyrosine kinases. Her2/neu overexpression is detected in about 10%–20% of Grades 2 and 3 endometrioid carcinoma [9, 18, 19]. These studies suggest that Her2/neu overexpression in endometrioid carcinoma characterizes late progression and differentiation events. *Her2/neu* overexpression is detected in approximately 9%–30% of serous carcinomas [20]. Elucidation of the role of Her2/neu in these pathogenic tumor types, therefore, requires further study.

### 2.3. *β*-Catenin

*β* *-catenin*, a component of the E-cadherin family of proteins, is essential for cell differentiation and maintenance of normal tissue architecture, and plays an important role in signal transduction. *β* *-catenin* also acts as a downstream transcriptional activator in the Wnt signal transduction pathway. A *β* *-catenin* mutation results in the stabilization of proteins that are degradation resistant, thus resulting in cytoplasmic and nuclear *β*-catenin accumulation and constitutive target gene activity. The accumulation of *β* *-catenin *is demonstrated by immunohistochemistry. Several studies have analyzed endometrial cancers, showing that nuclear accumulation of *β*-catenin is significantly more common in endometrioid lesions (31% to 47%) compared to nonendometrioid histologies (0% to 3%) [21]. In another report, *β*-catenin nuclear accumulation was more frequent in endometrial hyperplasias than in endometrial carcinoma samples, suggesting a *β*-catenin role in the early development of this tumor type [22]. In fact, alterations in *β*-catenin have been described in endometrial hyperplasia that contains squamous metaplasia or morules. Koul et al. found that all *β* *-catenin *mutated tumors were estrogen-receptor (ESR) positive and most were progesterone-receptor (PgR) positive, thus suggesting a dependence on estrogen stimulation during endometrial carcinogenesis [11]. In contrast, there is no correlation between *β* *-catenin *mutations and Microsatellite Instability (MI) or *K-ras* or *PTEN* mutations.

### 2.4. AKT

The phosphatidylinositol 3-kinase (PI3K) AKT pathway is activated in many human cancers and plays a key role in cell proliferation and survival. *PIK3CA* mutations frequently occur with other genetic alterations such as *Her2/neu*, *K-ras,* and *PTEN* in several types of tumors. Endometrial cancer is known to possess various genes alterations which activate the PI3K-AKT pathway. The frequency of mutations for *PIK3CA* in endometrial cancer is reported to be 28% [23]. However, Shoji et al. reported that *AKT1* (E17K) mutations were detected in 2 out of 89 tissue samples and 0 out of 12 cell lines [24]. They suggested that *AKT1* mutations might be mutually exclusive from other PI3K-AKT activating alterations, although *PIK3CA* mutations frequently coexist with other gene aberrations. Additional mutations in AKT family members in endometrial cancers were reported in *AKT2 *(D399N, 426T, and 141T) and in *AKT3* (E438D) [25]. Taken together, studies found that 5 out of 41 endometrial cancers have mutations in AKT family members at a frequency of approximately 12%.

### 2.5. FGFR2

Alterations in the fibroblast growth factor receptor 2 *(FGFR2)* gene causes the receptors to become active, leading to cell proliferation. Byron et al. reported mutations in FGFR2 in 10% of primary uterine tumor samples [26]. Mutations were observed in 16% of the endometrioid histology subtype tumors. In primary endometrioid endometrial cancers, *FGFR2* and *K-ras* mutations were mutually exclusive. Conversely, *FGFR2* mutations were seen together with *PTEN* loss-of-function mutations. The authors also showed that endometrial cancer cell lines with activating *FGFR2* mutations are selectively sensitive to the pan-FGFR inhibitor, PD173074 [27]. In addition, upregulation of FGF2 mRNA expression was observed in endometrial cancer specimens [28]. These data suggest that investigation of these agents may be therapeutically beneficial for endometrial cancer patients.

## 3. Loss-of-Function Genetic Events

### 3.1. PTEN

Endometrial carcinomas are characterized by a variety of genetic alterations, but the most frequent alteration is in the *PTEN *gene. *PTEN*, located at chromosome 10q23, encodes a protein and lipid phosphatase which behaves as a tumor suppressor gene. *PTEN *inactivation is induced by mutations that lead to a loss of expression and is induced to a lesser extent by a loss of heterozygosity. The PTEN protein has both lipid and protein phosphatase activities, with each serving different functions. The lipid phosphatase activity of *PTEN *induces cell cycle arrest at the G_1_/S checkpoint. In addition, the upregulation of proapoptotic mechanisms involving AKT-dependent mechanisms is mediated through *PTEN*, as is the downregulation of anti-apoptotic mechanisms through Bcl-2 [29–31]. *PTEN *further acts in opposition to PI3K to control levels of phosphorylated AKT [23, 32]. A PI3K mutation is seen in 36% of endometrioid endometrial cancers and is common in tumors that also carry the *PTEN *mutation. The protein phosphatase activity of *PTEN *is involved in the inhibition of focal adhesion formation, cell spread, and migration, as well as the inhibition of growth-factor-stimulated MAPK signaling [33]. The *PTEN* gene, which acts as a tumor suppressor gene, is present in individuals and causes increased cancer susceptibility, including those with Cowden's syndrome. *PTEN* mutations are the most frequent genetic lesions in endometrial adenocarcinomas of the endometrioid subtype. *PTEN* mutations are reported in 25%–83% of tumors, more frequently in endometrioid carcinomas and microsatellite unstable tumors, and are, thus, the most frequent genetic alteration reported in cancers [34]. *PTEN* gene alterations are associated with metastatic behavior and advanced stage in other cancer types. In contrast, the loss of PTEN function is an early event in endometrial tumorigenesis. Several groups have described a concordance between MI status and *PTEN* mutations; the mutations occur in 60%–86% of MI-positive endometrial carcinoma EEC cases, but only occur in 24%–35% of MI-negative tumors. Genetic alterations that account for *PTEN* protein inactivation include various mutations, a loss of heterozygosity (LOH), or promoter hypermethylation, with mutations occurring the most frequently [30]. *PTEN* promoter methylation is observed in 19% of cancers and is significantly associated with metastatic disease [35]. Kim et al. reported that *PTEN* and *K-ras* double-mutant mice (*Pten^d/d^K-ras^G12D^*) exhibited dramatically accelerated endometrial cancer development compared to cancers formed from a single *PTEN* or *K-ras* gene mutation [36]. These results suggest a synergistic effect of dysregulation of the *PTEN* and *K-ras* signaling pathways during endometrial tumorigenesis.

### 3.2. P53

The *p53* gene is located on chromosome 17 and is important in preventing the propagation of cells with damaged DNA. *p53* mutations or TP53 overexpression is twice as frequent in tumors without hyperplasia (estrogen unrelated) than in those with hyperplasia (estrogen related) [11, 37]. This is consistent with other data in which the most striking genetic alteration, present in about 90% of serous carcinomas (estrogen-unrelated NEEC), is a *p53* mutation [38]. In other reports, statistically significant correlations were observed between *p53* alterations and non-endometrioid histology type, high-grade tumors, and the absence of the progesterone receptor [39]. On the other hand, *p53* genetic alterations were observed in 17% of endometrioid carcinomas, which were primarily Grade 3 [40]. The exact mechanisms causing this mutation are still not well characterized. In response to DNA damage, nuclear *P53* accumulates and causes cell cycle arrest by inhibiting Cyclin D1 phosphorylation of the Rb gene and thereby promotes apoptosis. Therefore, mutated *P53* results in a nonfunctional protein that accumulates in the cell and acts as a dominant negative inhibitor of wild-type *P53*, leading to propagation of aberrant cells. *p53* mutations in endometrioid carcinoma are a late event during progression or differentiation. *P53* alterations play a relatively minor role in clear cell type endometrial carcinoma in comparison to the serous type [41]. *p53* mutations are also rarely observed in ovarian clear cell adenocarcinomas in comparison to endometrioid adenocarcinomas [42]. As a result, it is possible that the pathogenesis of clear cell carcinoma in the female genital tract arises from a unique pathway [43].

## 4. Genomic Instability

The most important types of genomic instability in endometrial cancers are MI and chromosomal aneuploidy. DNA mismatch repair (MMR) deficiency, detected as MI, is the most common molecular phenotype in endometrioid cancer, as *PTEN* tumor suppressor gene mutations. MI is seen in cancers (colonic, endometrial, and others) of patients with hereditary nonpolyposis colon cancer (HNPCC) and is also present in 28% of sporadic endometrioid cancers but is not present in serous cancers [40]. MI is distributed almost equally among the three histopathological tumor grades of endometrioid cancers. However, MI is rare in the clear cell type [44]. HNPCC patients with endometrial cancers have an inherited germline mutation in *MLH-1*, *MSH-2*, *MSH-6*, or *PMS-2*, but endometrial cancer only develops after the instauration of a deletion or mutation in the contralateral *MLH-1*, *MSH-2*, *MSH-6*, or *PMS-2* allele. Following this, the deficient MMR (MLH-1, MSH-2, MSH-6, or PMS-2) causes the acquisition of MI and the development of the tumor. Inactivation of the mismatch repair gene *MLH1* by methylation of the promoter seems to be the most frequent cause of MI in sporadic endometrioid carcinomas, followed by a loss of the expression of other two mismatch repair genes, the *MSH2* and *MSH6 *genes. The mechanism for the inactivation of *MSH2* is still not clear, as promoter methylation and mutations are rare. *MSH6* inactivation is usually caused by a mutation. 

Aneuploidy is frequent in serous cancers, and is uncommon in endometrioid cancer. When present, aneuploidy is exhibited predominantly by Grade 3 tumors [45, 46]. These data suggest that a different type of genomic instability is associated with the different histopathological-type tumors. However, in some reports, no significant correlations were found to exist with either the *K-ras* or *p53* mutations [7, 11, 47]. 

Telomeric attrition triggers genomic instability in certain cancer types. Both EEC and NEEC cells have short telomeres in endometrial cancer. However, only NEECs are significantly associated with critical telomere shortening compared to adjacent morphologically normal epithelium, thus suggesting that telomere shortening contributes to the initiation of NEECs but not EECs [48]. The authors also proposed a model in which telomere attrition gives rise to the initiation of NEECs and the progression of EECs.

## 5. Genetics Events outside the Cancer Pathway

Genetic variation acting either within or outside of the cancer cell may determine the outcome of interaction with exogenous or endogenous carcinogens. Endometrial stimulation by estrogens without the differentiating effects of the progestins is a primary etiologic factor associated with the development of endometrial hyperplasia and carcinoma [3]. There is evidence that estrogens and some of their metabolites are involved in the endometrial cancer pathogenesis. Estrogens and some of their derivatives are genotoxic and induce DNA damage, which if not removed could, thus, contribute to an increased risk of malignancy. Defects in the estrogen metabolism can result in defective apoptosis, DNA repair, and proliferation [49, 50]. Estrogens mediate their effects via the estrogen receptors (ESRs), estrogen receptor alpha (ESR1) and estrogen receptor beta (ESR2), which activate its metabolic pathways. The polymorphisms of ESR1 and ESR2 suggest an association with an increasing risk of developing endometrial cancer [51]. Cytochrome P450 1B1 CYP1B1 is a constitutively expressed and inducible enzyme with a central role in the oxidative metabolism of a wide range of endogenous and exogenous compounds including many carcinogens [52, 53]. Saini et al. reported that *CYP1B1* depletion in endometrial cancer cells leads to decreased cellular proliferation and induced G0-G1 cell cycle arrest, thus suggesting that CYP1B1 inhibition in endometrial cancer cells could be a useful therapeutic approach [54]. Progesterone or its synthetic form has been used as a primary treatment or palliative treatment of advanced and recurrence endometrial cancer, because progesterone inhibits estrogen-induced endometrial proliferation. In addition, the loss of progesterone-mediated Wnt signaling inhibition in the endometrium plays a rate-limiting role in tumor onset and progression [55].

## 6. Inherited Predisposition

### 6.1. Lynch Syndrome

Lynch syndrome, or hereditary non-polyposis colorectal cancer (HNPCC), is characterized by an increased risk for colorectal cancer. Endometrial cancer is the most common malignancy in patients with Lynch syndrome or HNPCC [56]. Lynch syndrome is caused by an inherited mutation in the MMR gene family, such as *MLH1*, *MSH2*, *MSH6*, *PMS1,* or *PMS2* [57]. The age at diagnosis of Lynch syndrome associated endometrial cancer is approximately 2 decades younger than that for sporadic endometrial cancers [58]. Parc et al. demonstrated that 34% of young patients with endometrial cancer (median age 46) were associated with MI, 57% of the MI positive group showed an absence of* hMLH1* expression, 19% showed an absence of *hMLH2* expression, and 23.8% demonstrated a normal expression of both proteins, while 9.5% of all patients were diagnosed with Lynch syndrome [59]. In another report, the development of the latter tumors of Lynch syndrome is significantly associated with MSH2/MSH6 protein complex deficiency [60].

### 6.2. Familial Site-Specific Endometrial Carcinoma

The clustering of endometrial carcinoma alone, termed as familial site-specific endometrial carcinoma, may constitute a separate entity. Eight percent of this group have been reported to have germline MMR mutations [61]. This mutation rate is lower than that of Lynch syndrome with endometrial cancer patients, of whom 15% show MMR mutations [62]. The difference in MMR, mutations, therefore suggests the existence of different genetic alteration pathways in familial site-specific endometrial carcinoma.

## 7. Malignant Mixed Mullerian Tumors (MMMTs)

Carcinosarcomas (malignant mixed mullerian tumors, or MMMTs) are currently excluded from uterine sarcoma and classified as metaplastic carcinoma, and many studies include these as NEECs [63]. However, endometrial carcinoma and MMMTs develop along distinctive molecular genetic pathways and exhibit different biological features. In MMMT,* p53* alterations occur early, during progression, just prior to clonal expansion and acquisition of genetic diversity [64]. In addition, changes in the AKT/*β*-catenin pathway may be essential for both the establishment and maintenance of phenotypic characteristics of MMMTs, playing key roles in the regulation of E-cadherin through transactivation of the *Slug* E-cadherin repressor gene [65]. Vaidya et al. reported that according to the discrepancy in survival the patients of MMMT should not be included in studies of endometrial cancers [66]. From this viewpoint, future studies will identify factors to classify these diseases.

## 8. De-Differentiation of Endometrioid Tumors

Mixed serous and endometrioid tumors have serous components that may be related to the “de-differentiation” of endometrioid tumors. This concept would explain the presence of overlapping EEC and NEEC features, both morphological and molecular in some tumors [67].

## 9. Epigenetic Changes

Aberrant CpG island hypermethylation in promoter regions occurs in many cancer-related genes, including those associated with cell cycle control, apoptosis, and DNA repair. Usually, unmethylated CpG islands become methylated, causing transcriptional silencing in cancer cells. Banno et al. reported that the frequencies of aberrant hypermethylation were 40.4% in *hMLH1*, 22% in *APC*, 14% in *E-cadherin*, and 2.3% in *R* *A* *R*-*β* in endometrial cancer specimens [68]. However, no aberrant DNA methylation was found in the *p16 *gene. Other genes inactivated by promoter hypermethylation in endometrial cancer include PgR [69], the cell cycle control genes 14-3-3 sigma [70], homeobox gene HOXA11, thrombospondin-2 gene (THBS2) [71], paternally expressed gene 3 (PEG3) [72], as well as the detoxifying enzyme glutathione S-transferase P1 (GSTP1) [73]. The impact of methylation on these genes in endometrial cancer development has not been well established. In endometrial cancers, differential DNA methylation patterns are detected in EICs and NEECs, suggesting divergent epigenetic backgrounds and unique tumorigenic pathways [74]. Promoter hypermethylation is a frequent event in EIC but not NEECs [75]. Many of the tumor suppressor pathways that are mutated in EIC can also be inactivated by hypermethylation.

## 10. The Future

The goal of screening endometrial cancers is to identify all patients who have a risk for developing this disease. Therefore clarification of the molecular and genetic mechanisms of development or progression of this disease is required. Understanding the genetic changes underlying cancer development or progression in the different histological subtypes is important for discovery of new targets for both diagnosis and therapy for individual patients.

## Figures and Tables

**Table 1:** Clinical and pathological features of endometrial carcinoma.

	Type I (EEC)	Type II (NEEC)
Age	Pre- and perimenopausal	Postmenopausal
Behavior	Stable	Progressive
Grade	Low	High
Hyperplasia-precursor	Present	Absent
Unopposed estrogen	Present	Absent
Myometrial invasion	Minimal	Deep
Specific Subtypes	Endometrioid carcinoma	Non-endometrioid carcinoma
Prevalence	70–80%	10–20%
Risk factors	Obesity, anovulation, nulliparity and exogenous estrogen exposure	In atropic endometrium

**Table 2:** Genetics features of endometrial carcinoma.

	EEC	NEEC
Gain-of Function		
*K-ras *	15–30%	0–5%
*Her2/neu *	10–20%	9–30%
*β* *-Catenin *	31–47%	0–3%
Loss-of Function		
*PTEN *	35–50%	10%
*P53 *	10–20%	90%
Genomic instability (microsatellite)	20–40%	0-5%
